# Who Takes Part in the Political Game? The Sex Work Governance Debate in Israel

**DOI:** 10.1007/s13178-020-00476-4

**Published:** 2020-07-21

**Authors:** Stephanie Levy-Aronovic, Yeela Lahav-Raz, Aviad Raz

**Affiliations:** 1 Department of Sociology and Anthropology, Ben Gurion University of the Negev, Beer Sheva, Israel; 2 School of Criminology, University of Leicester, Leicester, UK

**Keywords:** Sex work policy, Neo-abolitionist legislation, Israeli sex industry, Stigma, Agency

## Abstract

**Introduction:**

This study explores the recent neo-abolitionist legislation of the Israeli sex industry by illustrating the competing claims of various stakeholders: those leading the legal change and those protesting it. The main question is how Israeli sex workers perceive the public debate over governing the Israeli sex industry.

**Methods:**

This study combines qualitative methods that include ethnographic observations and interviews. The ethnographic observations were carried out between November 2018 and October 2019 in gatherings, protests, and academic conferences where sex workers were the lead speakers. In addition, 16 in-depth semi-structured interviews were conducted with sex workers across various indoor sectors, and four interviews were conducted with political figures to learn about their efforts to adopt neo-abolitionist legislation.

**Results:**

the Israeli legislative proceedings initiated in 2007 deny sex workers a voice and exclude them from the political space and policy debates that have a direct bearing on their working lives and wellbeing. Thus, Israeli sex workers perceive sex work governance as controlling their agency and deepening their stigmatization. In this process, we show how contrasting groups became strange bedfellows in their attempt to protect sex workers by incriminating clients of the sex industry.

**Conclusions:**

We conclude that the binary framings of debates about sex work in Israel do not address the actual needs or political desires of sex workers who are ignored and excluded from the discourse about them.

**Policy Implications:**

Furthermore, we conclude that the issue at hand is not about permitting sex workers to express their views but rather about the need to listen to their critiques to ensure that policy is built on their knowledge and experience.

## Introduction

On February 10, 2020, Israel’s State Attorney’s Office issued administrative closure orders for all the strip clubs in Tel Aviv under the assumption that these clubs are a form of commercial sexual exploitation and promote the sexist view of women as sex objects. The police subsequently raided these clubs and closed them for 30 days. This decision is an illustration of sex industry governance that has characterized Israel since 2007, as it leans toward the adoption of neo-abolitionist legislation by passing a series of laws, among them the “Prohibition on Consumption of Prostitution” which passed on December 31, 2018, and will come into force in July 2020.^1^ This legislation aims to eradicate different aspects of the sex industry under what Weitzer (2019) called “moralizing discourses.”

While it has been argued by many scholars (Anasti, 2017; Baratosy & Wendt, 2017; Fassi, 2015; Jeffreys, 2010; Oselin & Weitzer, 2013; Pitcher, 2019; Sanders & Campbell, 2014; Weitzer, 2012) that sex workers ought to be involved in the legislative process that directly affects them, Israeli sex workers were not initially included or invited to participate in the law reform process. By adopting the prostitution-as-harm narrative and “speaking on behalf” of sex workers (Lahav-Raz, 2019), the Israeli proceedings are similar to the process witnessed worldwide (Harrington, 2018; Huschke, 2017; O’Connell Davidson, 2003; Pitcher, 2019; Weitzer, 2007, 2019); namely, a coalition between the religious right and abolitionist feminists that have engaged in a heated debate over the realities of sex workers and appropriate sex work policy. These two parties have thus become strange bedfellows in an attempt to protect sex workers within the framing of a paradigm of oppression.

Joining the criticism of sex work scholars regarding the disregard of the voices of sex workers (Agustín, 2008; Armstrong, 2019; Pitcher, 2019; Sanders & Campbell, 2014; Weitzer, 2017; Yarbrough, 2020), this article addresses the question of how Israeli sex workers perceive the public debate over the governance of the sex industry in Israel. Based on fieldwork observations and interviews with both women working in the sex industry and legislators, we argue that the Israeli legislative proceedings initiated in 2007 discredited, silenced and excluded sex workers’ voice, thus, denying them parity of participation in democratic spaces which, as was shown by FitzGerald and McGarry (2016), perpetuates inequalities against an already disenfranchised group. Israeli sex workers, therefore, view state and neo-abolitionist discourses as controlling their agency and deepening their stigmatization.

Although this lack of state recognition impacts their lives and working conditions, we show that Israeli sex workers have, nonetheless, managed to generate solidarity and political-class consciousness as a basis for their collective action. Despite the powerful and “irreversible” nature of sex work stigmatization (Hardy & Cruz, 2019), they have managed to initiate various grassroots protests alongside social media activism, from which they gained the confidence to organize by establishing “Argaman,” the first Israeli sex worker organization. Their social struggle emerged when their right to recognition and dignity in the political process was violated. That is also when their motives for social resistance and rebellion have formed. When starting to share individual experiences, the feeling of disrespect became the motivational basis for their collective strength aiming to rebel against the laws while empowering them of the sense of moral and social worth. Thus, we conclude that even though the legislation passed, and sex workers might have been seen to lose the battle, their calling for prioritizing strategies to address women’s poverty and vulnerability to socioeconomic marginalization instead of seeing them as traumatized women with “false consciousness,” they stirred their own political-class consciousness, gained more vocal power, and carved a path into everyday public discourse.

In the following, we first provide an overview of the literature on the different models of sex work governance while focusing on the Israeli case. We then outline the methodology used in this study, before discussing the findings which demonstrate the nuances of sex workers’ perspectives. We conclude that the issue at hand is not about permitting sex workers to express their views but rather about the need to listen to their critiques in order to ensure that policy is not only built on their knowledge and experience but rather, as Dodsworth (2018) suggested, moves beyond polemical, binary discourses that have little connection to sex workers’ lives.

### Models of Sex Work Governance

The twenty-first century has seen renewed attention to prostitution and sexual commerce. According to Sanders, Brents, and Wakefield (2020), the global politics of borders and its concern with gender inequality and human rights have affected recent trends in policies on commercial sex, in which different groups battle out how sex should be governed in the modern age. There are three conventional policies regarding sex work governance. The first is the decriminalization of sex work, which most sex worker rights organizations worldwide are advocating in favor (for example, NSWP, Fuckförbundet, International Union of Sex Workers, ICRSE, and SWAN) as well as human rights organizations such as Amnesty International and the World Health Organization. Decriminalization is based on the sex-as-work narrative that perceives sex work as a profession. Thus, sex workers deserve the same rights as any other employee (Abel, 2014; Baratosy & Wendt, 2017; Fassi, 2015; Gira Grant, 2014; O’Connell Davidson, 2003; Oselin & Weitzer, 2013; Pitcher, 2015, 2019; Sanders & Campbell, 2014; Weitzer, 2017).

The second policy is a prohibitionist model that criminalizes all parties involved in sex work in an attempt to abolish the industry. Scholars (Anasti, 2017; Baratosy & Wendt, 2017; Jackson, 2019; O’Connell Davidson, 2003; Pitcher, 2019; Weitzer, 2009, 2018) have argued that full criminalization contravenes sex workers’ human rights, restricts their access to health services, and fosters stigma and discrimination. There is, they state, still no evidence that it is even possible to prevent the sale of sex. The third policy, which has received considerable support over the last two decades, is the “End-Demand” approach, which adopts the prostitution-as-harm narrative. The End-Demand policy is based on radical abolitionist feminism, which views sex work as a form of violence against women, inherently degrading, harmful, and exploitative (Oselin & Weitzer, 2013). This neo-abolitionist model pioneered by Sweden (1999) criminalizes male purchasers of sex and removes all laws that criminalize female sex workers. Intending to minimize gender inequality, it shifts the focus from the sellers to those they perceive as perpetrators (Halley, Kotiswaran, Shamir, & Thomas, 2006; Harrington, 2018; Matthews, 2018; Sanders et al., 2020; Vuolajärvi, 2019; Waltman, 2011). The Swedish neo-abolitionist law has now evolved into a global “supermodel” for prostitution policy (McGarry & FitzGerald, 2018; Hammond, 2015; Kingston & Thomas, 2018; Sanders & Campbell, 2014), and bills to criminalize clients have been passed in Iceland (2009), Norway (2009), Canada (2014), France (2015), Northern Ireland (2016), and, most recently, Israel (2018).

This model has, nonetheless, been subject to much criticism. For example, Kingston and Thomas (2018) argued that a coherent Nordic model is questionable, not least because the Nordic laws operate in different contexts and are not implemented in existing legislation or by practitioners in the same way. Policy and law have thus become, they claim, a mechanism for the transference of ideology and rhetoric. Other critics have focused on how the Nordic model can pose significant risks to sex workers’ safety by increasing resources of policing to their activities (Brooks-Gordon, 2006; Kingston & Thomas, 2018; Smith & Mac, 2018). Sex workers are invisible in this anti-prostitution crusade (McGarry & FitzGerald, 2018; Weitzer, 2007), precisely because their accounts clash with neo-abolitionist goals.

Notwithstanding, there is, as yet, no ideal legislative model for sex work. Although most sex workers led organizations are strongly in favor of decriminalization (Filar, 2020; Gira Grant, 2014; Jackson, 2019; Pitcher, 2015), Weitzer (2017) explained further that neither criminalization nor decriminalization has sufficient data to decide which model brings about better consequences. According to Shamir (Halley et al., 2006, 360), a close look at the legal regimes suggests that although each model influences sex work and sex trafficking differently, they are flawed in similar ways. Since all models presented are binary, focusing on either agency or victimhood, and their discourse rarely includes the voices of sex workers (Smith & Mac, 2018), there is a dire need to hear directly from sex workers affected by the legislation, preferably prior to making a final decision. This is also the case with the changes on sex work policy that have taken place in Israel since 2007 where, as in other areas (Hammond, 2015; Pitcher, 2019), the public and political discourse deny sex workers the opportunity to express their opinions about the legislation that directly concerns and affects them.

### The Israeli Sex Industry

The international migration of the Nordic model (Kingston & Thomas, 2018; Pitcher, 2015, 2019; Vuolajärvi, 2019) is also evident in Israel. Since 2007, various laws supporting the prostitution-as-harm narrative have been promoted by abolitionist-feminist MKs from both left-wing and right-wing parties in the Knesset, among them the criminalization of clients. On December 31, 2018, after almost 12 years of discussions, the law to incriminate sex industry clients was passed in the Knesset (due to come into force in July 2020). The toned-down version includes administrative fines with a voluntary alternative of participating in a preventive training course and no criminal record.

As part of the repeated hearings, the Knesset Subcommittee on Combating Trafficking in Women and Prostitution initiated a national survey to estimate the scope and characteristics of the Israeli sex industry. The survey, which serves to this day as the only available statistical information about Israel’s sex industry, was published in 2016 (Santo, Carmeli, & Rahav, 2016). Its findings estimated that more than 12,000 people were involved in prostitution, 95% of whom were women, and that the cumulative total of payments for sex work services in 2014 was 1.3 billion NIS. The findings of this survey have proved controversial with both legislators and sex workers, who have argued that the statistics are dubious and are used to uphold a particular ideological viewpoint.^2^ However, both parties use the data for their own needs.

According to Lahav-Raz (2019), the law to criminalize clients can be viewed as the success of determined efforts, originally initiated by MK Zehava Galon as a radical left-winger in the opposition, to advance the neo-abolitionist perspective and put an end to commercial sex. However, it was only when conservative right-wing parliamentarians in the coalition joined Galon’s cause that the bill got the attention needed to become a law. As in other places (Colosi, 2013; Harrington, 2018; Pitcher, 2019), these efforts were driven by “morality politics” dominated by an ideology of family values and by conservative, libertarian, and feminist political actors who claimed to be speaking “on behalf of” women in the sex industry and who argued that allowing the sex industry to exist freely is an abuse of human rights, harmful to all women, and should not be tolerated in Israel.

As mentioned earlier, Israeli sex workers were not initially included or invited to participate in the law reform process. As a consequence, they formed various social media protests and initiated TV interviews, demanding to be part of the discussions on the proposed law and for no legislation about them to be made without them (Lahav-Raz, 2019). Noticing their escalated protests, Knesset members approved sex workers’ restricted attendance at Knesset meetings. However, sex workers and their representatives were allocated only a few minutes per discussion, which was not enough time to represent their range of perspectives.^3^ Likewise, the legislative process was already in motion, and sex workers were, therefore, not able to make a real impact on the outcome.

In light of these events, this study examines how Israeli sex workers perceive the public debate over governing the sex industry in Israel and argues that they see sex work governance as controlling their agency and deepening their stigmatization. We present the process of governance in Israel alongside the two dichotomous discourses on sex work and the opinions of both legislators and sex workers on this topic. Furthermore, we claim that viewing the sex work industry through dichotomies and reductionist perceptions prevents sex workers from representing their own diverse experiences.

## Method

This study combines qualitative methods that include ethnographic observations and interviews. The ethnographic observations were carried out between November 2018 and October 2019 in gatherings, protests (such as the first strippers’ protest in Tel Aviv), and academic conferences where sex workers were the lead speakers. Six ethnographic observations were also conducted at different strip clubs in Tel Aviv during 2018.

In addition, 16 in-depth semi-structured interviews were conducted with sex workers across various indoor sectors in an attempt to capture their perspectives on the Israeli legislative process, and four interviews were conducted with political figures to learn about their efforts to adopt neo-abolitionist legislation. All the interviews were conducted between February 2018 and March 2019. The Ben-Gurion University Ethical Committee approved the research.

### Population and Participants

The participants comprised 16 Israeli women, six of whom were not originally from Israel, who stated that they had previously worked (seven participants) or were currently working (nine participants) in different indoor sectors of the Israeli sex industry (e.g., stripping, camming, escorts, discrete apartments, and BDSM). Their average age was 28.25 (range = 21 to 53), eight were married or in serious relationships, and five had children. The Israeli sex workers we interviewed hold diverse views regarding prostitution policy. Of the seven women who were no longer working in the industry, four supported the neo-abolitionist legislation process in Israel, while the other three opposed; of the nine women still working, all opposed the neo-abolitionist legislation.

All the interviews were conducted in Hebrew by the first author, and most were conducted face-to-face, with two taking place on the telephone, one over Skype, and one through email correspondence. We contacted interviewees by publishing a request on social media and then used the snowball method to proceed. The interviews were carried out at locations that facilitated a private conversation, such as coffee shops, participants’ living rooms, and outdoors, according to the participant’s request. The average time for an interview was 90 min, and all interviews started with an explanation of the purpose of the study, namely, to hear their opinions about legislation of the sex industry in Israel. The participants were assured that their identity would remain confidential, and they were instructed not to answer any questions they were uncomfortable with. Participants’ names were changed in the article to maintain anonymity.

In addition to interviewing sex workers, the first author also conducted four interviews with political figures from different sides of the political spectrum: Esq. Nitzan Kahana, head of the Task Force on Human Trafficking and Prostitution, MK Dr. Aliza Lavie, former chair of the Subcommittee on Combating Human Trafficking and Prostitution, MK Shuli Mualem, an active member of the Committee on the Status of Women and Gender Equality, who submitted (along with MK Zehava Galon) the bill that became the Prohibition on Consumption of Prostitution Law, and MK Michal Rozin, former executive director of the Association of Crisis Centers for Victims of Sexual Assault, who initiated the bill to close strip clubs and to define lap dancing as an act of prostitution.^4^ Two of these interviews were conducted face-to-face, while the other two were conducted over the telephone. The combination of voices, both from different sides of the political spectrum and from women inside and outside the sex industry, has contributed to a nuanced understanding of the compound perspectives on the sex industry and its legislation in Israel.

### Data Analysis

The data analysis was carried out by the first author under the close guidance of the second and third authors who are experienced qualitative researchers, following the stages suggested by Strauss and Corbin (1994). First, all the interviews were transcribed. The transcripts were then recoded into key categories, and similar categories were later merged into broader categories. Each category was analyzed separately to gain a complex and rich understanding of the participants’ experiences until a more comprehensive organizing framework was conceptualized to represent their experiences faithfully.

### Findings

#### “The greater good of women”

The debate over governing Israel’s sex industry, which was initiated in 2007, positioned feminist politicians and public figures directly against sex workers. On November 16, 2018, a panel called “The Sex Industry in Israel – Complexities, and Representations” was held by the Task Force on Human Trafficking and Prostitution with the participation of former sex workers and NGO members. The first author observed this panel whose purpose was to shed light on the intricacy of choosing sex work as a profession and the possible outcomes of legislation before its passing. During the discussion on the complexities of the industry, Professor Orit Kamir, a radical feminist and human rights activist, intervened to protest. Kamir (2003), who co-drafted the Israeli Prevention of Domestic Violence Law in 1991 and proposed a new conceptualization of sexual offenses including a draft bill that was endorsed and presented by three parliament members in 2003, claimed during the panel that “the law against sex work is for the greater good of women, even if it means we have to sacrifice the sex workers today for a better future.” Kamir’s words represent the popular neo-abolitionist, feminist perception in Israel that sex workers do not truly want to work in the sex industry. In addition, she asserted that sacrifice in the form of sex workers would “save” the status of Israeli women in general.

A similar perception was expressed in an interview with MK Michal Rozin, a left-wing opposition member who proposed the amendment “Prohibition on Offenses Related to Prostitution” in 2018^5^(i.e., banning lap dancing in strip clubs). During the interview, she described the social change she was looking to create as part of her values: “I am not trying to save anyone…I come from a feminist standpoint. I do not want this phenomenon to go on, just as I am opposed to slavery.” Rozin also addressed the sacrifice perception referred to by Kamir on the website of the Association of Rape Crisis Centers in Israel:

From a social perspective, the sex industry perpetuates the rape culture and deepens the gender inequality. I will not accept the reality that women and men sell their bodies and their sexuality for the sexual satisfaction of others. As a society, we have a responsibility to determine how we want to see ourselves as a whole. If this patronage leads to the eradication of the harm to women—I am willing to take full responsibility (Rozin, 2018).

Rozin’s words, which resonate with general public opinion and the opinions of other MKs, promote her core values in the name of all women in Israel. Likewise, MK Dr. Aliza Lavie, a religious right-wing coalition member and former chair of the Subcommittee on Combating Human Trafficking and Prostitution, explained in her interview: “The law puts forward our feminist opinions and our responsibility toward society. It portrays my values as well as government values.” MK Shuli Mualem, a religious right-wing coalition member who joined MK Zehava Galon in bringing the governance discourse to life in parliament, said “I fought for the law, first and foremost, because of my Jewish and personal value set. It is firstly a moral law and only secondly a feminist law.”

These examples show how the religious and feminist Knesset members, despite other ideological differences, largely agree on the issue of eradicating sex work. As was demonstrated by Weitzer (2007, 2019) in the case of the moral crusade in the USA and by Huschke (2017) on the debate about sex work in Northern Ireland, the single-issue focus of neo-abolitionist, feminist groups on the sex industry trumps all other issues. It explains their willingness to work with right-wing religious groups in order to legitimize their efforts as a bipartisan enterprise. Furthermore, Israeli MKs use a moralizing discourse that is motivated by altruistic and humanitarian objectives and has both symbolic and instrumental goals.

Concerning the symbolic aspect of the law, while the left-wing abolitionist feminists tried to endorse a universal value set against the commodification of sex and the female body, the religious right-wing MKs supported a more religion-based value set, attempting to bolster Jewish moral standards rooted in protecting women’s modesty and maintaining the traditional family structure. As to the instrumental goal of the law, the purpose was to criminalize clients and provide aid for women working in the industry who are now seen as victims, prostituted women, sex slaves, or survivors. This is despite evidence that a victim narrative deprives sex workers of their agency (Armstrong, 2019; Brown & Sanders, 2016; Pitcher, 2019) and that “risk discourses” function to exclude so-called non-normative and deviant activities such as sex work (Sanders, 2006). Israeli lawmakers’ broad generalization of sex workers as victims creates, according to Brooks (2020), a binary dichotomy between good and evil; generalizations are therefore inappropriate and exclude sex workers from this discourse. The following section thus focuses on how Israeli sex workers view the change in Israel’s sex work policy. We show how their views, which contradict non-sex working feminists, are being erased from the public discourse under the presumption that they are deluded and not worthy of a voice.

#### “Nothing about us without us”

Since the start of the legislative process in 2007, there has been no involvement or representation of currently working sex workers in the legal proceedings. However, in recent years, sex workers started expressing their growing frustration at being excluded and erased from the discourse that has produced knowledge about them without their involvement. Via social media platforms that achieved extensive public exposure (Lahav-Raz, 2019), they succeeded in forcing politicians to invite them to parliamentary hearings on the issue.

Their principal demand of “nothing about us without us” became a rallying call against the knowledge and authority narrative. They demanded to be involved in the process for two reasons: first, a sense that their agency was being stripped away since their voices and opinions regarding a law that will affect them directly was not heard; and second, a concern that the law will increase their stigmatization and, as has happened in other places, forcing them to go further underground (Andrade et al., 2019; Huschke, 2017; O’Connell Davidson, 2003).

Lior, a former sex worker, described her frustration at not being considered when legislation was being decided on her behalf: “My voice has the significance of a little girl who doesn’t know what’s good for her…you won’t tell me what it will do to me.” Lior, who supports Israeli sex workers through her Facebook page and personal correspondences, echoed her own and others’ frustrations with being treated like women who are not worthy of voicing their opinions and who cannot differentiate between good and evil and therefore need the help of those who can. In other words, sex workers have become subordinate to the knowledge made about them by elite voices of legislators and other outsiders (Yarbrough, 2020). They are, thus, pushed aside and deemed as objects unworthy of voicing their various opinions (Agustín, 2008; Pitcher, 2015, 2019).

As part of this study, we presented both legislators and sex workers with each other’s views. One of the themes expressed by the former in their interviews was their genuine concern for women working in the sex industry. We asked Hila, a stripper and sex worker activist who recently discussed sex work on national television, for her opinion about the legislators’ claims of governing in the name of caring. She answered:

In my opinion, if you care for someone, as they claim to, then they should come and talk to us. We’re not children. They need to listen to my experience and try and help me without dictating their views to me.

Both Hila and Lior used the analogy of children versus adults to describe the feeling of belittlement. In many of our interviews, both former and current sex workers described the legislation process as taking place “over their heads” and deciding for them what is best for them, which, they stated, was “not very feminist” nor a show of genuine concern. In light of this belittlement, they asserted that their inclusion in the parliamentary discussion would have led to the creation of a more inclusive and comprehensive governance law. There is no doubt that the nature of sex workers’ experiences addresses an essential dimension of the complexity of sex work—a complexity that neither radical feminism nor sex work advocates have adequately theorized (Barton, 2002; Dodsworth, 2018). Yet, nothing illustrates the complexity of women’s sexual agency more vividly than the real lives of sex workers.

Of the women we interviewed, most described the legislative process as a reduction of their agency, as propaganda that expands social stigma, and as destructive for women working in the industry. They reported attempts to take part in legislation meetings but, when finally attending, being allocated no more than a few minutes to speak. It demonstrates Fraser’s (2010) claim about frame-setting tactics that determine who is included/excluded from the universe of those entitled to consideration in matters of distribution, recognition, and political representation. Silver, a sex work activist, described her experience of attending a parliamentary meeting:

One of the things we felt was that no matter how much we spoke, it was only for their protocol, it was for them to say “we heard you” and then set us aside…We got no more than five minutes to speak.

MKs and other involved parties who have been associated with the feminist activity are, according to sex workers, performing an anti-feminist act by not involving sex workers in the discourse about them. On the other hand, for a radical abolitionist feminist such as Kahana, feminism means true equality for women. Thus, she expresses the perception that “if one woman is for sale, it sends a message that all women are potentially for sale”:

There can be no equality for women as long as there is prostitution. It just cannot happen. You can be a prime minister, and if the people sitting in your government are consumers of the sex industry, then, to them, you are a potential prostitute.

Kahana believes that criminalization laws are helpful for those women who want to leave the industry; namely, the women she wants to help:

I am not trying to prevent sex workers from working in the sex industry because they are potentially hurting me. I am interested in 76% of women who admitted to the national survey that they want to quit the industry.

Kahana presents both the radical abolitionist, feminist narrative and what Flaherty (2016, as cited in Yarbrough, 2020, p. 69) called a “savior complex” narrative, according to which relatively privileged outsiders ignore the definitions of problems and solutions by the groups of people most directly affected and, instead, impose their own will. This narrative can also be destructive to sex workers’ agency, as the use of statistics designed to portray that outsiders “know what’s best” and have better suggestions than those actually working in the industry (Pitcher, 2019).

It has been shown that sex workers are rarely included in research exploring how sex work should be governed (Aroney & Crofts, 2019; Baratosy & Wendt, 2017; Pitcher, 2015, 2019) and are thus silenced and marginalized (Brown & Sanders, 2016; Colosi, 2013) which may result in short-lived governance (Sanders & Campbell, 2014; Weitzer, 2018). The call by Israeli sex workers for laws to be made in cooperation with them may be a solution; it is not, however, equitable to other marginalized social groups who receive their verdict from rules made on their behalf. In the next section, we elaborate on the implications of the legislative process in Israel and the binary discourse portrayed by the elite voices, which have further stigmatized and marginalized sex workers.

### Deepening Stigma and Eradicating Agency

According to Gira Grant (2014), the universal “whore-stigma” is one of the reasons why it has been so hard for sex workers to generate political-class consciousness. At the same time, it is one of the foundational contributions of sex worker feminists to feminist discourse and activism: challenging whore stigma in the name of all those who live under it. This objective is also at the center of the Israeli sex workers’ activities. For example, on May 3, 2018, a protest was held in a central area of Tel Aviv, against Rozin’s bill to ban lap dancing from strip clubs, which views lap dancing as an act of prostitution and strip clubs in general as a form of commercial sexual exploitation of vulnerable women. The first two authors attended the protest, where the protesters were holding signs and yelling slogans such as “My body, my business” and about the potential outcomes such as “stigma kills.” Hila, a stripper for the past 4 years and one of the protest organizers, explained the reason for the protest and the platform she was trying to create:

The protest forged a platform for women who are being silenced, breaking the stigma about them. Up until now, we were considered women who are not worthy of voicing our opinions, so we proved that we are by exercising our right to protest.

In 2019, a year after the first protest, Hila came out on public television about her work as a stripper and her advocacy for sex-as-work in an attempt to “break a few stigmas” and “open people’s minds to the nuanced opinions about sex work legislation made without consulting them.” She subsequently experienced varied responses ranging from a harsh backlash on social media to her plea to decriminalize sex work to expressions of sympathy and support. By the time of the second protest in 2020, it was evident that many other sex workers were following in her footsteps, as the number of women protesting grew considerably, and the media did not hesitate to report on their call to reopen the strip clubs.

Hila was not the first to come out of the “sex work closet.” Sarah is a former sex worker who came out in public a few years before Hila and was recently interviewed about her experiences by a leading newspaper in Israel.^6^ Sarah, who defines herself as a ‘survivor of prostitution,’ presents a narrative of how agency and victimhood can coexist, which is more complicated than the prevailing binary discourses suggest. In her interview, she explained that she considers herself an advocate of the neo-abolitionist perception regarding eradicating the sex industry. Nonetheless, she has taken a leading role in Argaman and acknowledges that the stigmatization of current and former sex workers is exacerbated by silencing them:

I agree that the feminist discourse around sex work legislation worsens the stigma about us. It does not allow for any complexity. Instead, it maintains a one-dimensional discourse in which you won’t be heard if you don’t take part. None of us takes part in this political game because we all know it doesn’t serve anyone in our community [sex workers]. It only serves those who play the political game—politicians, pimps, and feminist activists. They play and rule the game.

Sarah described a combination of actors who have power in and over the sex industry. The actors who play this political game are all beneficiaries of the legislation, albeit on different sides: politicians and feminist activists can reinforce the elite voices legislating on behalf of sex workers (Aroney & Crofts, 2019; Yarbrough, 2020), while third-parties can benefit from the growing underground market of sex work that may emerge after legislation (Agustín, 2008; Huschke, 2017; Kilvington, Day, & Ward, 2001; O’Connell Davidson, 2003; Vuolajärvi, 2019).

We noticed in our interviews that sex workers allied around a joint mission of preventing the deepening of already challenging stigma. This stigma is twofold: first, the well-known universal “whore stigma” of sex work (Armstrong, 2019; Gira Grant, 2014; Hardy & Cruz, 2019; Sanders, 2005; Weitzer, 2018), which the Nordic Model aimed to pass from the sellers to consumers who now perceived as the perpetrators; and second, the stigma created by legislation advocates who refer to sex workers as “victims” (Armstrong, 2019; Bettio, Della Giusta, & Di Tommaso, 2017; Filar, 2020; Huschke, 2017; Pitcher, 2019; Weitzer, 2019). For most sex workers, it is not the actual work that causes the most harm; rather, it is the stigma attached to earning an income through sex work in a context where it is criminalized and being justified for their degradation (Gira Grant, 2014; Huschke, 2017). Sarah outlined an additional aspect of this stigma:

[They treat us as] those strippers who are a bunch of rape victims and could not do anything better with their lives and their trauma, so they became strippers. They treat us as if we are pawns in the service of patriarchy, and their goal is to prevent other women from getting raped.

This stigma is created by the discourse of legislators who call sex workers victims and see them as traumatized pawns who need saving. While it is clear that legislators are not deliberately trying to create another layer of stigma, they are, however, trying to depict sex work as something that can only be done to victims. This approach creates an additional layer of stigmatization that may further prevent sex workers from coming out in public or seeking help if needed and demonstrates the deflection of the stigma back on to sex workers.

This additional layer of stigma can also be regarded as the re-stigmatization of sex workers as women with false consciousness or as victims with no agency of their own (Huschke, 2017; Phipps, 2017). Lior described how sex workers’ agency is obliterated when legislation is discussed without considering their voices: “You are erasing my presence from the discourse. It erases my agency; it erases everything. Even before having workers’ rights, this is, first and foremost, my identity.” Lior portrayed feeling unseen and unworthy of being heard; her presence as a human being and personal identity is eliminated when she is excluded from the discussion about the type of work she and others have chosen. According to Baratosy and Wendt (2017), it is not necessarily the discourse but the criminalization of sex work that perpetuates the stigma of sex work and consequently marginalizes sex workers. Others (Brown & Sanders, 2016; Huschke, 2017; Vuolajärvi, 2019) observed that the stigma and public shame attached to commercial sex (as perpetuated by the dominant views expressed in the policy debate) degrade sex workers and make them feel victimized and less connected to society. For Lior, this is a direct outcome of the law:

Stigma is one of the things that weaken sex workers. We [Argaman] along with other sex workers’ organizations aim to increase public awareness towards it since the sigma is too heavy [for them to do so on their own]. The bottom line is that legislation deepens stigma.

In line with many scholars (Agustín, 2008; Andrade et al., 2019; Brown & Sanders, 2016; Colosi, 2013; Fuckförbundet, 2019; Pitcher, 2015; Vuolajärvi, 2019), Lior suggested that the neo-abolitionist legislation could lead to increased stigmatization in Israel as happened in Sweden after the introduction of the Sex Purchase Act in 1999. Yet, neo-abolition is not the only form of legislation that has been found to increase stigma, as was argued by Weitzer (2009), who claimed that all types of legislation may feasibly increase stigma. The neo-abolitionist legislators do not, arguably, mean to exacerbate an already severe stigma. Despite the intention of the Nordic Model to pass the stigma from the sellers to the perpetrators, many Israeli sex workers we interviewed argued that legislation would most likely create, if it is not already there, a new layer of stigmatization that forces them to deal with even more social criticism and pushes them further to the sidelines of society.

Our findings are thus in line with Fassi’s (2015), and Armstrong’s (2019) claims that social stigma makes sex workers’ lives less stable, less safe, and far riskier. Moreover, Israeli sex workers’ voices have been systematically silenced by actors whose social, political, and economic capital entitles them to speak and act on behalf of sex workers on the basis that sex workers are powerless victims or deviant perpetrators. In other words, it is not the fact that a person exchanges sexual services for money that is oppressive; rather, oppression comes from the fact that this person is culturally and legally marginalized and denigrated.

It should be noted that the law to criminalize sex industry clients in Israel will only come into force in July 2020. It is, therefore, still too early to assess the actual effect of legislation inspired by the Nordic Model on sex workers and their clients in Israel. However, it should be remembered that simply adopting a model from another country because it appears to work does not, as Kingston and Thomas (2018) showed, consider wider social issues and, therefore, may not work in another country. Furthermore, as was demonstrated by Fuckförbundet (2019) in Sweden and by others (Armstrong, 2019; Bettio et al., 2017; Huschke, 2017) elsewhere, the legislation to criminalize clients has led to sex workers being increasingly stigmatized, hunted by the police, and disempowered. In other words, the state is becoming a primary source of violence and exploitation of sex workers (Hammond & Attwood, 2015). These findings should be considered, and such outcomes avoided when the legislation in Israel comes into effect.

## Discussion

By focusing on the recent legal change and its aftermath as a constitutive media event in the public discourse on sex work in Israel, this study illustrated the competing claims of various stakeholders: those leading the legal change and those protesting it. The analysis of the political stakeholders showed how, in the attempt to create a new sex work policy in the name of caring, neo-abolitionist feminists had been encouraged to forge alliances with those they would usually view as “enemies” of feminism (O’Connell Davidson, 2003). These contrasting groups have become strange bedfellows in an attempt to protect sex workers by incriminating the clients of the sex industry and passing several laws aiming to eradicate the industry as a whole.

The first part of the analysis demonstrated the uniqueness of the Israeli scene by highlighting the intersecting agendas of the secular abolitionist feminist left-wing and the religious right-wing as well as the joined efforts of coalition and opposition. The Israeli case shows how legislators in Israel pursued the goal of gender equality by putting aside their political differences and framing their desire via the victim narrative of sex workers. In other words, even though they sit on both sides of the political map and hold different opinions on religion and feminism, MKs came together to legislate against sex industry clients in the fight against commercial sex. They thereby took on the role of speaking on behalf of sex workers based on the claim that sex workers are victims that must be saved. This demonstrates the argument put forth by Filar (2020) that sex workers are not allowed to write their “herstories” nor do they have the power to self-memorialize or to determine whose actions are worth remembering, whose voice is worth hearing.

Furthermore, the binary framings of debates about sex work in Israel—focusing on choice versus force—do not address the actual needs or political desires of sex workers. In fact, they do sex workers a political disservice and may also influence the dichotomy, or false dichotomies (Bettio et al., 2017), between supporting and opposing the legislation. This policy thus serves to marginalize further and stigmatize sex workers, which is part of a process of creating binary distinctions between sex workers and others (Colosi, 2013).

The second part of our analysis focused on how Israeli sex workers perceive the public debate over governing the sex industry in Israel as controlling their agency and increasing their stigmatization. Since prostitution policy is often located within “violence against women” strategies nationally and locally (Pitcher, 2015), it may not be surprising that sex workers are against sex governance. However, our findings demonstrated the nuances of their protests and grassroots activism, which sparked a form of political-class consciousness among protesting sex workers. None of the women we interviewed, including those who self-identify as ‘survivors of prostitution,’ had conformed to a victim identity. Thus, their sparked political-class consciousness revolves around seeing oneself as an actor and in control of one’s life, and not as a passive victim. Furthermore, in all of their protests and grassroots activism, they call for harm-reduction support, not rescue. For labor rights and decriminalization, not criminalization, harassment, and violence.

Concerning the increase of stigmatization, our findings showed that the heated public debate on sex work policy, as well as the victim narrative adopted by MKs, prevented sex workers from asserting their choice to work in the industry. Scholars (Bettio et al., 2017; Huschke, 2017; Pitcher, 2019) have argued that the more sex work is stigmatized, the less agency sex workers are granted. Moreover, alongside the victim narrative, Israeli sex workers are often treated as “children” needing to be saved, protected, and told what is best for them. As children, they are excluded, pushed aside, and deemed unfit to voice their opinions. This is against all evidence (McGarry & FitzGerald, 2018; Pitcher, 2015), showing the importance of positioning sex workers as central in debates on the future of their industry as a way of ensuring that policy formation is built on their knowledge and experience of diverse working practices rather than on moral campaigns.

## Conclusions

Although the Israeli effort to eradicate the sex industry, beginning in 2007, culminating in the passing of the law to incriminate sex industry clients and banning lap dancing, we conclude that the regulation of the sex industry cannot be evaluated solely on the sole basis of success or failure. While peripheral, the Israeli case study demonstrates sex workers’ ability to protest against governmentality by building bottom-down solidarity and developing political-class consciousness.

In contrast to Weitzer’s (2018) claim that sex workers lack solidarity and channels to mainstream media, we highlight how, despite the thick stigma of sex work, Israeli sex workers have managed to sound their opinions at an unprecedented volume. Although it is impossible to define a “typical” sex work experience (Majic, 2018), nor one “sex worker voice” or one sex worker identity (Dodsworth, 2018), Israeli sex workers outspoken disagreement and differences as both individuals and as a group (through the organization of Argaman), demonstrate the ability to challenge the feminist canon by building a community of cultural advocacy while protesting their delegitimization.

For example, just recently, on June 29, 2020, a follow-up hearing was held in the Constitution and Law Commission for the implementation of the law to incriminate sex industry clients. Prior to the hearing, Argaman, alongside four other organizations,^7^ drafted a position paper, endorsed by social workers and Jurists, as well as by “Israel Women’s Network,” calling for amending the law and postpone it for 24 months. They called legislators to avoid further harm to sex workers and survivors of prostitution who are in financial and mental distress, especially due to the COVID-19 pandemic. Moreover, postponing the law will enable the authorities and aid organizations to assess and provide solutions for the populations expected to be most affected by the law, following the various needs in employment, welfare, housing, and health. The claimes of Argaman and its allied organizations resonate with Smith and Mac’s (2018) argument regarding how contemporary feminists’ disapproval of prostitution remains unmoored from pragmatism. Hence, more political energy goes to obstructing sex work than to what is really needed, such as helping sex workers avoid prosecution, or ensuring viable alternative livelihoods that are more than respectable drudgery.

In conclusion, by listening to Israeli sex workers’ perceptions of their experiences, we aimed to contribute to the development of a ground-up politics of justice that has meaning for them. We thus, join many sex work scholars who assert the dire need to create a more inclusive model of social justice for sex workers based on a discourse of rights and recognition. This model should not only “allow” sex workers to express their views; it should, as Majic (2018) claimed, center sex workers as producers of knowledge.

## Footnotes

1 Prohibition on Consumption of Prostitution, 2019

2 For example: https://www.facebook.com/sexworkersspeak/posts/332010317315329

3 For further reading, see: *The Constitution*, *Law*, *and Justice Committee*. (2018). Protocol meeting No. 710.

4 Penal Law Amendment, 2018

5 https://main.knesset.gov.il/Activity/Legislation/Laws/Pages/LawBill.aspx?t=lawsuggestionssearch&lawitemid=2064915

6 A citation is not provided in order to protect her identity.

7 “Gila project for trans empowerment”; “ma’avarim:Trans Community Empowerment”; “Trans Israel”; and “Ahoti—for Women in Israel”.
